# Spectrum and antibiotic resistance of catheter-associated urinary tract infections

**DOI:** 10.3205/id000032

**Published:** 2017-11-22

**Authors:** Béla Köves, András Magyar, Peter Tenke

**Affiliations:** 1 Department of Urology, South-Pest Hospital, Budapest, Hungary

## Abstract

Catheter associated urinary tract infections (CAUTIs) are amongst the most common nosocomial infections and are also considered among the most common complications associated with indwelling urinary catheters. Most catheter associated infections are derived from the patient’s own perineal flora, however the presence of a catheter increases the chance of being colonised by cross transmission of nosocomial bacteria as well. Most episodes of short-term catheter-associated bacteriuria are asymptomatic and are caused by single organisms, while long-term catheterisation promotes multibacterial infections and colonization. With prolonged duration of catheterization bacteriuria is considered universal because of the formation of biofilms on the surface of the catheter. Chronic indwelling catheters are an important reservoir of different multiresistant gram-negative organisms, therefore they are frequently isolated from CAUTIs. Treatment of catheter associated asymptomatic bacteriuria is not recommended because it will only promote the emergence of resistant organisms without effectively clearing the urine of catheterised patients.

## Summary of recommendations

Regarding bacterial spectrum and antimicrobal resistance of catheter associated urinary tract infections the following recommendations should be highlighted:

- Routine urine culture in asymptomatic catheterised patients is not recommended (Gr: B).
- Treatment of catheter associated asymptomatic bacteriuria is not recommended (Gr: A).

## 1 Introduction

Urinary tract infections (UTIs) represent at least 40% of all hospital acquired infections with the majority of cases being catheter associated [1], [2]. In patients without catheters microorganisms that ascend from the urethra are usually of enteric origin (e.g. *Escherichia coli* and other *Enterobacteriaceae)*. However the presence of a catheter creates a special environment for bacterial colonisation and biofilm formation, which increases the chance of being colonised by non-enteric nosocomial bacteria, like *Pseudomonas aeruginosa* as well. Therefore it is important to be familiar with the different spectrum of pathogens associated with the presence of an urinary catheter.

## 2 Methods

We performed a literature search in the PubMed database from 1970 to 2016 regarding the spectrum and antibiotic resistance of catheter-associated urinary tract infections (CAUTIs) using the following keywords in different combinations: catheter, urinary tract infection, bacteriuria, antibiotic resistance. Only publications written in English were selected. A total of 38 publications were identified through the search. Furthermore, data and recommendations of the European Association of Urology [3], the European Centre for Disease Prevention, Control and Centers for Disease Control and Prevention, including Guideline for Prevention of Catheter-associated Urinary Tract Infections from 2009 and recommendations for Healthcare-associated Infections (HAIs) were also collected. The recommendations were based on the level of evidence and the grade of recommendation. For this purpose the system modified from the Oxford Centre for Evidence-based Medicine was used [4].

## 3 Results

### 3.1 Routes and incidence of colonisation

#### 3.1.1 Pathogenesis

Transurethral ascent of microorganisms is the most common mechanism of UTI development, which provides a logical explanation for the increased risk of infection following bladder catheterisation or instrumentation. Bacteria can ascend through the lumen of the catheters by reflux of urine from the contaminated bags (intraluminal route) or along the extraluminal catheter-urethral surface. At the time of catheter insertion up to 20% of patients will be colonized immediately [5], [6]. Catheterised patients with catheter associated bacteriuria develop bacteremia in 0.4–4% of cases. In acute care facilities catheter associated UTI is one of the most common causes of bacteremia [7], [8], [9], [10] because of the high frequency of catheterisation. For *closed catheter systems* the incidence of bacterial colonisation is increased by 3–8% with each day [11], therefore colonisation is considered universal by the end of the month. The development of bacteriuria is universal within 3–4 days in case of catheters with *open-drainage systems*. Impairment of the natural defense mechanisms (e.g. obstruction, immunsuppresion), leads to reduction of virulence requirements of any bacterial strain to induce infection.

#### 3.1.2 Biofilm

Biofilm formation is a universally occuring phenomen on the surface of the catheters in the urinary tract. Biofilms are structured communities of microorganisms encapsulated within a self-developed polymeric matrix adherent to a surface [12]. These bacteria may differ from their planktonic free-floating counterparts in many aspects, such as metabolic rates or antibiotic susceptibility. The formation of biofilms on catheter surfaces is the reason why bacteriuria becomes universal in case of long-term catheterisation. 

### 3.2 Microorganisms and spectrum 

#### 3.2.1 Short-term catheterisation

Short-term (<30 days) [13] catheter-associated bacteriuria is generally caused by **single organisms** and is asymptomatic. However it may be polymicrobial in up to 15% of cases [10], [14]. During initial catheter insertion or catheter exchange, transient asymptomatic bacteraemia is common in chronically catheterised patients [15]. The risk of bacteraemia during initial catheter insertion may be similar, whether there is a pre-existing UTI (7%) or sterile urine (8.2%) [16], [17] (IIa). The incidence of febrile UTI and bacteraemia is relatively low since colonisation of urethral catheters is caused mainly by less virulent organisms and a non-obstructed catheter effectively drains the infection. The bacterial spectrum reflects the locally prevailing flora (eg. community, hospital). According to the European and Asian guidelines on management and prevention of catheter-associated urinary tract infections the most frequently occurring bacteria during short-term catheterisation are *E. coli*, *Pseudomonas aeruginosa*, *Klebsiella pneumoniae*, *Proteus mirabilis*, *Staphylococcus epidermidis*, *Enterococcus* spp. and *Candida* spp. [10].

Pyuria, which however varies by organism, has no diagnostic value, and is present in most cases of catheter-associated bacteriuria.

#### 3.2.2 Long-term catheterisation

Indwelling catheterisation lasting 30 days or longer [13] is defined as ‘long-term’ or ‘chronic’. With prolonged duration of catheterisation biofilm formation on the catheter surface occurs universally [18], [19], [20]. As a consequence microorganisms may acquire increased resistance against antimicrobials and become almost impossible to eradicate without removing the catheter. In long-term catheterised patients **polymicrobial** bacteriuria occures in up to 95% of the cases, with usually 3–5 isolated organisms. During long-term catheterisation, the commonest bacteria is *E. coli*. Other associated flora include *Providencia stuartii*, *Pseudomonas*, *Proteus*, *Morganella* and *Acinetobacter*, *Enterococcus* and *Candida* spp. [10].

While the catheter remains in situ, the spectrum of free-floating microorganisms and bacteria in the biofilms shows a dynamic turnover. Bergqvist et al. found that one-quarter of the samples obtained by an indwelling catheter did not show complete agreement with the bladder aspiration samples suggesting that some organisms may colonise the catheter only [21].

#### 3.2.3 Occuring bacteria

Similarly to the bacterial spectrum of uncomplicated UTIs, *E. coli* is the most common pathogen in the presence of a catheter as well. However in the presence of a catheter we find higher proportions of other bacteria, especially a higher rate of gram-positive pathogens. 

The National Healthcare Safety Network (NHSN) is a national surveillance system for patient and healthcare personnel safety in the USA. It aims to surveil selected HAI data at locations other than intensive care units, in hospitals and other types of healthcare facilities. In hospitals reporting to the NHSN between 2006–2007, the most frequent pathogens associated with the presence of an indwelling catheter were *E. coli* (21.4%) and *Candida* spp. (21.0%), followed by *Enterococcus* spp. (14.9%), *P. aeruginosa* (10.0%), *K. pneumoniae* (7.7%), and *Enterobacter* spp. (4.1%) and *Acinetobacter baumannii* (1,2%). A smaller proportion was caused by other gram-negative bacteria and *Staphylococcus* spp. [22]. Moreover at one US tertiary care academic centre *Enterococcus* spp. (28.4%) and *Candida* spp. (19.7%) were reported to be the most common pathogens [9], [23].

The persistence of *E. coli* strains is related to the presence of Type 1 pili, an adhesin for uroepithelium as well as the Tamm-Horsfall protein. Colonising *E. coli* strains lack P fimbriae in most cases of catheter-associated infections [24].

*Enterococcus* species, especially *Enterococcus faecalis* and *Enterococcus faecium* are among the leading causes of hospital-acquired UTIs [22], [25]. Many enterococcal isolates can produce biofilms. Catheter implantation results bladder inflammation and causes fibrinogen release and accumulation onto the catheter. *E. faecalis* takes advantage of the presence of fibrinogen and uses it as a resource through the production of proteases [26].

*P. mirabilis* is an organism of unique importance for CAUTIs. It is not typical in patients undergoing short term catheterization [27], however the longer a catheter is in place the more likely *P. mirabilis* will be present. It was found in about 40% of urine samples collected from patients with chronic indwelling catheters [9]. *P. mirabilis* has a uniquely strong biofilm forming activity compared to other uropathogens [28], and it is also a very potent urease producer. *P. mirabilis* hydrolyzes urea several times faster than other pathogens with urease activity [29]. Organisms producing urease may cause a crystalline biofilm [30], [31], which is similar to struvite stones, and it is frequently associated with catheter encrustation and obstruction [32]. Other urease producing species include *P. aeruginosa*, *K. pneumoniae*, *Morganella morganii*, other *Proteus* species, some *Providencia* spp. and some strains of *Staphylococcus aureus* and coagulase negative *staphylococci* [33]. 

Patients with urinary catheter also have an increased risk of UTI due to *Pseudomonas* spp. [8]. *P. aeruginosa* is an opportunistic human pathogen, which causes infections through biofilm formation on the surface of indwelling catheters. It utilizes a distinct mechanism to form biofilms, independent of exopolysaccharides during CAUTIs [34].

Another organism rarely found outside of the catheterised urinary tract is *Providencia stuartii* [20]. For this organism, the adhesins MR/K are more common [35], [36]. *Acinetobacter* is a group of bacteria commonly found in soil and water. Outbreaks of *Acinetobacter* infections including occasionally urinary tract infections typically occur healthcare settings treating very ill patients and rarely occur outside of healthcare settings [37].

*Candida albicans* readily causes a clinical UTI via the haematogenous route, but this is also can cause ascending infection if an indwelling catheter is present, or following antibiotic therapy [10]. 

Candiduria develops in 3%–32% of patients with short-term catheterisation [13]. In case of long-term catheterization the incidence of candiduria was 17% in a single study reporting on a population of individuals with spinal cord injury or multiple sclerosis [38]. The US NHSN no longer counts candida CAUTI in its surveillance definitions.

### 3.3 Antimicrobial resistance in the presence of a catheter

The increasing antimicrobial resistance against different antimicrobials is a common problem with urinary pathogens. Chronic indwelling catheters are an important reservoir of different multiresistant gram-negative organisms, such as extended spectrum beta-lactamase (ESBL) producing *Enterobacteriaceae* [39] or carbapenem-resistant *Enterobacteriaceae* (CRE) [40]. Therefore multiresistant organisms are often causes of CAUTIs.

One of the most important factors leading to increasing bacterial resistance is the fact that patients with indwelling urinary catheters often receive antimicrobials, usually for indications other than urinary tract infection [9]. Due to this intense antimicrobial exposure, antimicrobial resistant bacteria are frequently isolated from catheterised urine samples. Conversely, pathogens colonizing the drainage bags of catheterized patients were found to be a source for outbreaks of resistant organisms in acute care facilities [13], [41]. The urine of residents with chronic indwelling catheters was found to be the most common site of isolation of resistant gram-negative organisms in nursing home setting as well [39], [42]. Consequently, current data do not support the treatment of catheter associated asymptomatic bacteriuria because it will only promote the emergence of resistant organisms [43], [44] without effectively clearing the urine of catheterised patients.

In the annual summary of data reported to the National Healthcare Safety Network at the Centers for Disease Control and Prevention (2006–2007) 24,8% of *E. coli* isolates and 33,8% of *P. aeruginosa* isolates from CAUTI cases were fluoroquinolone-resistant. Against ceftriaxone, resistances of *E. coli* and *K. pneumoniae* were 5,5% and 21,2%, respectively. Resistance rates were relatively high even against carbapenems in the presence of a catheter: *E. coli* 4%, *K. pneumoniae* 10%, *P. aeruginosa* 25% and *A. baumannii* 25,6%. Significant resistance was found against vancomycin (6,1%) and ampicillin (3,1%) in case of *E. faecalis* as well.

## 4 Further research

Further research has to be aimed to develop antimicrobial agents that are also effective against bacteria enclosed in the biofilms. Creating an ideal catheter surface modification which can resist bacterial adhesion and biofilm formation in long-term catheterisation is also a challenge of the future.

## 5 Conclusion

Urinary catheters have long been recognized as major risk factor for developing healthcare associated UTIs. There is different spectrum of causative pathogens in the presence of a catheter due to biofilm formation and different resistance patterns from what we see in the urinary tract without a foreign body. Pathogens within the biofilm are well protected from antibiotics and from the host defense. Traditional microbiologic laboratory testing can detect planktonic free-floating bacteria within the urine, but pathogens within the biofilm will not be easily detected with routine methods. Since antimicrobal therapy in catheterised patients induces bacterial resistance and will be associated with the development of multi-resistant organisms, prudent antibiotic administration strategy, catheter management and prevention of CAUTIs should be considered high priority. Treatment of asymptomatic bacteriuria in catheterized patients should be avoided as this can only aggravate the problem of antimicrobial resistance in healthcare.

## Note

This article is also to be published as a chapter of the Living Handbook „Urogenital Infections and Inflammations“ [45].

## Competing interests

The authors declare that they have no competing interests.
